# *Umma and* the nation-state: dilemmas in refuge ethics

**DOI:** 10.1186/s41018-022-00124-z

**Published:** 2022-08-05

**Authors:** Hossameldeen Mohammed, Ray Jureidini

**Affiliations:** 1 Theology Faculty, Karabuk University, Karabuk, Turkey; 2 Research Center for Islamic Legislation and Ethics, Hamad Bin Khalifa University, Doha, Qatar

**Keywords:** Nation-state, *Umma*, Refugee-policy, Islamic ethics, Zakat, Forced migration, Refugees, Asylum, Border policies

## Abstract

This article addresses the question of the Muslim *umma* (the universal community of Muslims) which arises within the context of the ongoing debates about the status of refugees and their rights around the world, especially in the Muslim world, where a significant majority of migrants and refugees originate and are hosted. We question the existence of the Muslim *umma* under contemporary circumstances and discuss the extent to which the modern nation-state has undermined the principles of Muslim unity and solidarity beyond national borders. We also examine how Islamic ethics and law (*fiqh*) identify the imperatives of providing comfort and protection for refugees (whether they are Muslim or non-Muslim) individuals, groups, and communities when required or requested. We see this in principle, but not always in practice. Are refugee policies applied by Muslim-majority countries today in accordance with the fundamental Islamic teachings and experiences of the original *hijra*? Or have such principles been made redundant by the political-geographic imperatives of ethnicized nation-states? To what extent can applied policies in Muslim-majority countries be developed and enhanced in the light of *umma* principles that might alleviate the longstanding refugee crises in this troubled area of the world?

The article consists of three main sections. The first provides a theoretical and religious analysis of the idea of the *umma* as an inclusive entity that encompasses all Muslims, contrasted with the modern nation-state as an exclusive geographically defined political institution. The second looks at three Muslim-majority countries that played a significant hosting role in refugee crises over the past decade, namely Jordan, Bangladesh, and Turkey. We offer some insight in understanding to what extent their policies or behavior were based upon *ummatic* principles, or secularist notions of national interest and humanitarian aid? The last section summarizes the recent innovation providing for Muslim refugees through a UNHCR arrangement and adaptation with the Islamic charitable obligation of *Zakat*. It is suggested that the UNHCR Zakat Fund is an *ummatic* manifestation that has already proved honorable and viable. The conclusion calls for a better understanding of the *umma* as it is in the Islamic consciousness towards an effective reforming of refugee policies and practices, suggesting a further enhancement of *ummatic* institutional collaboration between the OIC and UNHCR for refugee integration.

## Introduction

Significantly, more than 67% of the world’s refugees originate from five Muslim countries. Syria, due to its ongoing civil war, remains the country of origin with the highest number of refugees (more than 6.7 million), accounting for almost one third of the world’s total refugee population. The second highest of 5.4 million refugees originate from Palestine. This is followed by Afghanistan (2.7 million), Myanmar (1.1 million) (this is not a Muslim-majority country, but the displaced Rohingya from Myanmar are Muslim), and Somalia (0.9 million) (UNHCR 2018, 3). The majority of the displaced are hosted by neighboring Muslim countries—Turkey (3.7 million—host of the largest number for the fifth consecutive year), Pakistan (1.4 million), Lebanon^1^ (998,900–the largest relative to its population), Iran (979,400), Bangladesh (932,200) and Jordan (691,000) (UNHCR 2018).^2^ Some non-Muslim countries also have accepted refugees, particularly Germany taking around 970,400 mostly Syrians (UNHCR 2019). Other countries have taken relatively few, such as the USA (18,000), Canada (40,000), and Australia (12,000) (Jureidini and Reda 2017). However, a significant proportion of refugees who are resettled in Islamic countries live under miserable circumstances. The majority of the host countries are less developed countries, suffering fundamentally from a lack of financial resources, and still continuing to bear the brunt of the global refugee crisis. According to UNHCR, in 2016 developing countries were shouldering 84% of the world’s refugee burden, and they did not receive any adequate financial support from other countries (UNHCR 2016). This puts a heavy burden on economies struggling for growth with little additional resources to deal effectively with such huge numbers of refugees.

890,000 Rohingya have been encamped in Bangladesh, at Cox’s Bazar, an area formerly known as a top tourist destination, particularly for newlyweds, but now offering a different reality. The Deputy Director-General of the World Health Organization, Peter Salama, revisiting the Kotobalong refugee camp in Cox’s Bazar, described the tough conditions he experienced: “The word that comes to mind when I just look around at this site of these makeshift houses is the word fragility. This is one of the most fragile situations I have ever seen, from a human perspective, from an ecological perspective, the word fragility just seems to sum it up.”^3^ Zayd Raʿd al-Ḥussayn, the UN High Commissioner for Human Rights at that time stated it was “a textbook example of ethnic cleansing” (Nebehay and Lewis 2017).

The Jordanian population consists of 25% refugees. Most Syrian refugees in Jordan also have difficult living conditions and over 80% of them live below the poverty line; among them, 51% are children, and 4% are elderly.^4^ There is no denial of the collaborative efforts of donor organizations, such as UN agencies, international and national NGOs, community-based organizations, and the government of Jordan in their bid to provide for refugees with essential needs, but UNHCR still sees these as insufficient (UNHCR 2016).

In Turkey, most refugees who were granted asylum are not allowed to work, even though they have good educational qualifications and labor skills. For example, since the Regulation on Work Permits for Foreigners under Temporary Protection was adopted in January 2016 until the end of 2018, only around 28,000 work permits were issued to adult Syrian refugees (19–64 years of age), comprising less than 1% of their population.^5^ In 2018, policy changes regarding Afghan refugees in Turkey (leaving decision making to the provincial directorates) resulted in a 92.5% reduction in the acceptance of International Protection status (ICMPD 2021). Thus, it is not surprising that many continued the still treacherous journey to Europe to earn a livelihood.

A different reality seems to exist in the wealthier Gulf States. They enjoy high rates of GDP, a strong infrastructure and plenty of capacity to incorporate migrants. Although they were accused of having taken “zero refugees,” critics did not consider that these countries are not signatories of the UN 1951 Refugee Convention and its 1967 Protocol. This means that there is no classification or registration of Syrians as “refugees” in GCC population statistics, hence they are not immediately identifiable. Various claims were made on the number of post-2011 Syrians taken in by the GCC states; for example, the Saudi Arabian government claimed it had 2.5 million Syrians in the Kingdom, most having arrived after 2011 (*Arab News* 12/09/2015),^6^ and the UAE stated it had taken over 100,000 Syrians after 2011 (*Khaleej Times,* 12/09/2015).^7^ Later in 2015, a study of publicly available data conducted by De BelAir (2015) concluded that, since 2011, the intake of Syrians amounted to a minimum of 420,000 in Saudi Arabia, 136,000 in UAE, 18,114 in Kuwait and 2,500 in Bahrain. Qatar’s claim of taking in 40,000 Syrians was unverifiable by De BelAir but was thought by the Syrian embassy in Doha to be around 27,000 (personal communication). By September 2015 it was recognized that Syrians could apply for either a work or tourist visa in the GCC, although it was costly and difficult. It was easier for those with family members in the Gulf to gain entry (Fathalla 2015). The trajectory continued to be Europe (mostly through Turkey) that prompted a warning by the UN High Commissioner for Refugees, António Guterres, that “Thousands of refugee parents are risking the lives of their children on unsafe smuggling boats primarily because they have no other choice” (Fleming 2015).

These particular conditions raise difficult questions more generally. To what extent has the notion of the Islamic *umma* and its teaching of mutual assistance been invoked to relieve the crisis of refugees? Has this principle of solidarity been affected by the secular culture of the modern nation-state? If the Muslim *umma* was operating today, would people from Muslim countries in crisis be compelled to risk their lives to seek shelter in Europe to be safe? Assuming some level of humanitarian concern is the practice of accepting refugees in Muslim countries associated more with secular international conventions, special political-economic motivations, and international pressure, rather than an *ummatic* association?

In this article, we briefly examine the transformation of *umma* that occurred after the emergence of the modern nation-state and how that has aggravated the crises for Muslim refugees. The examination of the *umma* concept does not seek to overlook the religious and ethical values that are inherent in it. Conversely, it aims to discover the implications of the absence or existence of religious and ethical meanings in refugee policies to develop a deeper perception of the reasons that lie behind the contemporaneous Middle East refugees’ problems. It suggests an ancient but enduring Islamic consciousness to address the dilemmas resulting from the Westphalia paradigm of the modern nation-state. Note, however, that drawing on the benefits of religious meaning does not come into contradiction with human rights, social solidarity, mutual respect, and other fundamental secular humanitarian values. On the contrary, it falls into what Wilkinson (2020,12) calls “secular-religious dynamics” where the secular and religious are in constant connection and engagement with each other to improve the humanitarian response to disasters. Put simply, this will help the international organizations to achieve better understanding of the religious beliefs and practices when they are responding to human catastrophes, albeit as secular organizations. As Wilson argued:

The secular and the religious shape and define one another, so that what is considered secular is affected by what is considered religious. Secular realms are increasingly influenced and shaped by ideas from the religious, just as religious spheres and actors are increasingly influenced and shaped by ideas from the secular. (Wilson, 2014, p. 235)

Our approach is threefold. The first provides a theological and sociological discussion of the idea of the *umma* as an inclusive meaning contrasted with the modern nation-state as an exclusive entity formulated by the colonialist powers that triggered competition and conflict between countries of the Middle East and the Islamic world in general. The second looks at three Muslim-majority countries that played a significant hosting role in refugee crises over the past decade, namely Jordan, Bangladesh, and Turkey. We offer some insight in understanding to what extent their policies or behavior were based upon *ummatic* principles or secularist notions of national interest and humanitarian aid. The last section summarizes the recent innovation providing for Muslim refugees through a UNHCR arrangement and adaptation with the Islamic charitable obligation of *Zakat*.

### The umma as a linguistic and Islamic concept

The primary meaning of *umma* in Arabic refers to a community of members who have certain ties and relations with each other, regardless of the type of these ties and relations; or, those who have certain characteristics or circumstances (Al-Iṣfahāni 2009, 87; Asad 2016, 177). The word *umma* appears in the Qur’an around 60 times, with multiple and diverse meanings, but commonly designated to those whom God has sent a prophet (Dallal, 2009, 775). That is *umma* is used in the Qurʾan to refer to all believers of God’s heavenly messages throughout history from the era of Adam to the mission of prophet Muḥammad (Q 21: 92). Further, the Qurʾan uses the notion of *umma* to refer to any group of people who have the same aim, or even other living beings, that have a certain association or common traits. Accordingly, the Qurʾan gives the name of *umma* to those people who gathered at a well for retrieving water.^8^*Umma* is also used to describe individual species of animals or birds.^9^ The Qurʾan sometimes uses the word *umma* to refer to other meanings in a metaphorical sense (Al-Ᾱlūsī 1994, 483). For example, Abraham has been described as an *umma*, “because the reference to Abraham also includes his sons Ishmael and Isaac, his grandson Jacob, and Jacob’s sons” (Nasr 2017, 1217).

It is generally understood, however, that the Muslim *umma* in the Qurʾan refers to those people who “believe in the guidance of One Allāh (Qurʾan) and prophethood of Muḥammad” wherever they are (Al-Ahsan 1992, 16). The term in the Sunna (prophetic tradition) has been used to signify the same meanings as in the Qur’an, but additionally involved broader meanings in some contexts.^10^ For example, it has been reported in Sahih Muslim, the most authentic book of Hadith after Sahih Al-Bukhari, that the Prophet considered Jews and Christians among members of the *umma*: “By Him in Whose hand is the life of Muhammad, he who amongst the *umma*, Jew or a Christian, hears about me ….” (Sahih Muslim, 1/134). Hallaq (2013, 42) considered the *umma* to be a supranational formation governed by moral and legal rules.

### Muslim *Umma* concept between inclusivity and exclusivity

The term Muslim *umma* has undergone significant transformations ranging from an exclusive religious connotation to inclusive overtones (social, cultural, and political). Both Islamic scriptures and the interpretations provided by jurists have contributed to diverse connotations of *umma* as a concept (Ali, 2002, 38). Although the Muslim *umma* is a religious phenomenon, it has been expanded politically to include non-Muslims who freely choose to live with Muslims in the same land, even without believing or accepting Islam, particularly Jews and Christians. Thus, from a political perspective *umma* refers to those people who share Islamic values, culture, and civilization and are residing permanently in an Islamic state.

Muslim jurists from different jurisprudential schools have unanimously agreed that all minorities who live in a Muslim society are members of the Abode of Islam (*dār al-islām* (Al-Sarkhasī 1989, 138). This legal position has been repeatedly confirmed by classical and contemporary scholars (Ansārī 2013, 501; Zaydān 2014, 91). In this tradition, Sheikh al-Qaradawi, notes: “Belonging to the Islamic *umma* accommodates Muslims and non-Muslims. They are all from Dar al-Islam, as the jurists decided” (al-Qaradawi, 1995, 18). This was confirmed in *ṣafīḥat madīna* (The Madina Charter or Constitution of Madina) in Articles 1, 25, and 26 (Denny 1977, 40–41; Lecker 2004, 29). The ultimate significance of this document is that it demonstrated how the state, from the Islamic perspective, was designed as a tool in the service of the *umma*, not the other way around (Al-Barghouti 2008, 61).

As noted by Denny (1977, 43), “All the kinship groups mentioned are subsumed under this *umma* idea.” Thus, “if the Jews are allowed to practice their own religion within the one *umma*, then this *umma* is no longer a religious community in the exclusive Muslim sense” (ibid., 44). Going further, Montgomery Watt concluded, “As they are specifically allowed to practice their own religion; however, this suggests that the *umma* is no longer a purely religious community” (Watt 1987, 268). The political inclusion of those minorities under the framework of a united *umma* was clearly due to their solidarity with the Muslims in defending the community (*jamāʿa*) against its enemies and their loyal commitment to this community as a whole, over any conflicting loyalties.

Contemporary scholars, however, disagree as to whether non-Muslim minorities are included within the *umma* or under what conditions (see, e.g., Watt 1987, 268; Al-Ahsan 1992, 21; Al-Barghouti 2008, 62). Saeed (1999: 308), For example, Hourani notes that during the early Islamic caliphate, “the treatment that non-Muslims received varied to a certain extent from caliph to caliph and depended on a particular caliph’s temperament and preference.” Under the caliph Umar, for instance, “the *umma* was to be based on Islam” and converts were to be given preferential treatment. At the same time, what is indisputable is that minorities under the umbrella of the *umma* were always considered as members of the Abode of Islam, where they enjoyed civil rights including moving from one place to another (except Mecca for religious considerations) and to stay anywhere they wished, like Muslims (Hourani, 2013, 4). Furthermore, Muslims were commanded to care of all minorities. Prophet Mohammed said:

If anyone wrongs a man with whom a covenant has been made [i.e., a dhimmi], or curtails any right of his, or imposes on him more than he can bear, or takes anything from him without his ready agreement, I shall be his adversary on the Day of Resurrection.(Abu Dawud, Vol4, Book34, # 3052)

It was also made clear that the responsibility of safeguarding their life and dignity falls upon the Muslims. Failing in this regard would bring God’s wrath:

Whoever killed a Mu’ahid (a person who is granted the pledge of protection by the Muslims, [i.e., a dhimmi] shall not smell the fragrance of Paradise though its fragrance can be smelt at a distance of forty years [of traveling].(Bukhari, Vol. 9, Book 83, #49)

The twelfth-century rabbi, Benjamin of Tudela (who started his journeying in 1165; around 560 Hijri) wrote about Constantinople (present-day Istanbul), then the center of the still-powerful Byzantine Empire. Local Jews he observed were pariahs and forced to live unprotected outside the city walls.^11^ But, as Haines (n.d.) points out, “not all medieval societies were so oppressive towards Jews, and in some places, Jews enjoyed considerably more freedom than they did in Christian-ruled lands.” By contrast, in Baghdad, the center of the “Abbasid caliphate, some 40,000 Jews lived free and easy, according to Benjamin. There were twenty-eight impressively opulent synagogues spread across the city and Jewish officials and institutions were treated with respect. “This sense of dignity and civility towards Jews was what stood out to Benjamin as the defining feature of Jewish life in the heart of Islam” (Haines n.d.).

Another good example of the social inclusion of minorities maintaining their own identities has been shown in the Ottoman millet system (Rassam 2003, 232). Garcia-Arenal points out that by the late sixteenth century (10th AH), all “non-Muslims were fully integrated into Ottoman society … by what is known as the millet system” which refers to relatively autonomous legal and religious communities within the religious pluralism of the Ottoman Empire. The millet system lasted until Western colonialism and nationalism made their momentous appearance (García-Arenal 2011, 603–604). Thus, “In the nineteenth century, however, under the influence of European nationalism and with grave implications for traditional arrangements, millet came to mean “nation” as well as “religious community” (Peterson, 2003, 340). Other researchers have confirmed that these minorities, under the description of *umma*, had enjoyed privileged circumstances compared to minorities living in other communities: “non-Muslims enjoyed considerable autonomy in personal-status law, worship, and education; they formed largely self-contained units with separate religious, legal, social, educational, and charitable institutions” (Kramer et. al. 1995).

This is not to say that minorities, including Jews, Christians, and polytheists (mushrikūn), were in an ideal situation in the *umma*. It should be remarked that there has been no ideal situation for any minority under majority rule anywhere, including contemporary times. Modern scholarship has swung between academics stigmatizing an *ummatic* system as oppressive and intolerant towards minorities (Bat Yeʾor, 1965, 36), to others arguing that there is no “inherent policy of humiliation towards non-Muslims in Islam” (Levy-Rubin, 2011). The assumption that historically the *umma* was quite exclusionary or discriminatory needs to be re-thought. All these contrasting viewpoints are important in shaping the inclusive and intercultural paradigm that should accommodate religious minorities in the *umma* of today and secure high standards in protecting their human rights.

Finally, it should be noted that relying on the religious texts, especially the provisions of Islamic law, to conclude that minorities in Islam have faced persecution and discrimination is not quite accurate. First, because these texts, just like any other religious texts, are made up of different views and notions that are open to different interpretations. Second, the actual practice towards minorities in most times was not confined to these texts, but was influenced by the economic and political context of the historical period, no different from today. Third, the judgment of these texts, which mirror the atmosphere of medieval societies to modern standards of human rights, is not entirely correct, because all religions and political systems prevailing in former times were accepting discrimination in one way or another. This is perfectly illustrated by Milka Levy-Rubin:

In ancient and medieval societies social hierarchy, as well as discrimination between various groups, was accepted, and was almost self-evident. (This is true, by the way, even for democratic Athens, where women, metoikoi, and slaves, who together made up the major part of Athenian society, did not have the same rights as the Athenian citizens and were socially inferior.) None of these societies believed in equality or equal rights the way modern Western societies do. (Levy-Rubin, 2011, 7)

### Umma and the universality beyond borders

All those people who lived in the Abode of Islam (*dār al-islām*), either Muslims or non-Muslims were able to live and move wherever they wished in a large area which represented the geographical boundaries of *umma* in pre-modern times. In his book, *The History of the Decline and Fall of the Roman Empire*, Edward Gibbon masterfully described its expansion:

Under the last of the Ommiades, the Arabian empire extended two hundred days’ journey from east to west, from the confines of Tartary and India to the shores of the Atlantic Ocean. And if we retrench the sleeve of the robe, as it is styled by their writers, the long and narrow province of Africa, the solid and compact dominion from Fargana to Aden, from Tarsus to Surat, will spread on every side to the measure of four or five months of the march of a caravan. (Gibbon 2004, 2295)

Even with the differences in faith between Sunnis and Shiites (the largest two branches of Muslims),^12^ which occurred early in the history of Islam, or even with the fragmentation of the original Islamic caliphate (main political authority) from the third *hijrī* century onwards (into the Abbasid caliphate in Baghdad, the Umayyad caliphate in Andalusia and the Fatimid caliphate in Egypt) or later wherein there were three large Islamic empires—Ottoman, Persian, and Moghol—the sense of belonging to the *umma* remained present and effective for most of history. Those Muslims and non-Muslims who settled in the *umma* land remained perfectly free to move between different regions and emirates and to reside wherever they desired without requiring any prior permission from the authority of their new abode. Although there were differences among the populations of these regions, the predominant trend was the integration of different ethnicities and the assimilation of incoming population flows (Hodgson 1974, 192–94). Mohammed Amara, the famous contemporary Egyptian thinker, suggested that this inclusive notion of the *umma*, which was stabilized within Islamic political thought from the very beginning of its history until the modern period, can be presented through the idea of Islam as a homeland—one *dār* (house) for one body of people. This bond could not be torn apart by nationality (as in the West) nor by the privileges of different nationalities (Amāra 1993). Thus, Sayyid Mawdūdī (1903–1979) observed that:

Differences on the basis of nationality, race and tribal conflicts did crop up now and again ... But the idea that the Muslims of the world constitute one *umma* remained intact [...]. A Muslim from any part of the world could go to any Muslim land without any restrictions, move freely in that country, stay there as long as he wished, engage in any trade, secure the highest government post in that country, get married without any difficulty. Islamic history is replete with instances where a Muslim went out of his country and lived in other Muslim lands for decades. He might have studied in one country, engaged in business in another, become a minister or commander-in-chief of the army in a third one and, then he might go over to yet another, settle there and get married. A well-known example is Ibn Baṭṭūṭa [d. 1377] who traveled through different Muslim countries for twenty-seven years. He did not need a passport or a visa to go to any of these countries. Nowhere was he questioned about his nationality. Nowhere did he face any difficulty in earning his living. He needed no permit to visit any place nor was any period fixed for his stay. If he sought a job under any government, he got appointed without any difficulty. (Mawdūdī, in Ahmad 1967, 14-15)

According to this *ummatic* tradition, the caliphate was not limited to a particular region. Even the capital of the caliphate shifted many times from one city to another—Damascus, Baghdad, and Istanbul (Hodgson 1974). Imām Muḥammad ʿAbduh (1849–1905), the Mufti of the Egyptian state, received the following question:

Is the Muslim who entered an Islamic kingdom considered one of its citizens, and does he have the same rights and obligations as them? Is nationality permissible according to Islam? Do capitulations^13^ exist among the Islamic, similar to what existed for foreigners in the Ottoman Empire?

The Mufti replied:

The Muslim homeland of the Islamic countries is the place where he intends to stay. There is no consideration to his birth or the country where he grew up in. As for nationality, it is not known in Islam, but nationality in European nations is similar to what was called by Arabs ʿ*aṣabīyya* and Islam came to abolish that ʿ*aṣabīyya* and erase its effects. (‘Amāra 1993, 505–508)

Here, therefore, is a religious leader explicitly rejecting the boundary limitations of the nation-state in favor of a universalistic Islamic *umma*. Thus, the notion of the Muslim *umma* constituted a supranational identity that precluded the classification of people on the basis of nationality or ethnicity. The consciousness of Muslims with regard to the *umma* first and foremost created a sense of universal fraternity. It rejected giving primary loyalty to ethnicity and nationalism, by transcending specific borders and boundaries that create political and identity separation between Muslims. This idealistic phenomenon, however, began to unravel from the late nineteenth to the early tweentieth century.

### The legacy of colonialism: contradictions between umma and nation-state

In this section, we will discuss two intertwined dimensions: first, the historical impact of colonialism on the *umma* that created long-lasting political, territorial, and ethnic conflicts in the region that are connected to the regional refugee flows today; and second, the continued existence of nation-states that maintain a colonialist formula in the Muslim world. Despite the heralding of the so-called post-colonial era, the nation-state system has restricted the potentiality of the global *umma* to the borders of modern nation-states and the policies that relate to migration and asylum-seeking across these borders.

### Umma and the legacy of colonialism

The current situation of the *umma* cannot be properly understood without tracing the historical context of colonialism and its implications on the *umma*. The colonization of the Muslim World began with the rise of the European empires. The first phase commenced with the British conquest of India followed by the scramble between the European empires for Africa in the nineteenth century. The last phase was the occupation of Arab lands of the Ottoman Empire after the First World War. The colonial era ended in the wake of the Second World War when Britain and France withdrew from most of their colonized territories (Esposito 1999, 552). However, the span of colonialism was not a mere historical stage in the life of the *umma*. The *umma* is not synonymous with empire.

It is noteworthy to mention that the colonial behavior did not come to an end. It is still vivid in some countries, especially Africa, and is still one of the major causes of refugee tragedies.^14^

According to Payind and McClimans (2016, 76), Islamic countries under colonial rule were reincarnated as they began to rebuild after sustaining the impacts of war, famine, and cultural hegemony. Economic collapse, war, forced migration, and famine carved new contours into the region we now know as the Middle East and created new cultural identities.

A significant part of the colonial re-shaping of the *umma* was the territorial demarcation by the British-French agreement dividing the Middle East, the Sykes-Picot Agreement. This put the nation-state model into practice, further deepened the ethnic divide and prevented the success of various unification attempts. The Syrian-Egyptian unity pact of 1958–1961, for example, proved to be a bitter failure.

Enormous efforts were made by the imperial powers to impose the Sykes-Picot nation-state. With the strategy of divide and rule, they deliberately undermined the unity of the *umma* through artificial boundaries, without consideration for the familial, tribal, cultural, or religious ties that differentiated them. They herded into these borders people who often had little to do with one another, with different languages, traditions, and cultures^15^ (Chomsky 2015, Falk 2015). Colonial authorities drew boundaries and sought to retain their control over the colonized by encouraging competition between ethnic, linguistic, religious, or tribal groupings. The territorial division of Muslim lands went hand in hand with national confusion and resulted in continual feuds over the right of ownership of some territories such as the Iran-Iraq dispute over “the *Shaṭṭ Al-ʿArab*, Pakistan and Afghanistan over the Durrand line; Pakistan and India over Kashmir; Saudi Arabia and Qatar, and Saudi Arabia and the United Arab Emirates, over borderline oases and oil fields; Libya and Chad over their border regions; and Iran and the United Arab Emirates over the Tunbs and Abu Musa islands” among other manifestations of colonial nation-state building (Esposito 1999, 552).

The competition between ethnic, tribal, and religious groups continues to raise severe problems in relation to migrants and refugees, such as xenophobia and racism. More importantly, it has managed to undermine the idea of a united *umma*. As Cole and Kandiyoti (2002, 198) conclude:

The continuing importance of the colonial legacy to the shaping of the modern nation raises severe problems of identity in the context of a nationalism that most often depicts itself as a pure nativism.

This explains the contemporary disunity of the *umma*. Sayyid, a professor of decolonial thought, suggests that the fragmentation of the *umma* has made it anachronistic*.* He proposed an alternate contemporary identification of the *umma* as a “Muslim diaspora,” finding it more useful in referring to “an empirical situation in which settler communities are relocated from their ordinary homes” (Sayyid 2014, 105). Here, we see the conceptualization of the nation-state^16^ eradicating the conceptualization of the *umma*, but where Muslim diaspora has not received any real purchase. *Umma* does seem to be an identity that is so all-encompassing, longstanding, and deep-seated that the idea of diaspora seems rather shallow by comparison.

It would, however, be wrong to assert that the Muslim world was politically united before the advent of colonialism and its state paradigm. It is not fair to say that all fragmentation and problems that characterize the *umma* today is simply a result of the nation-state and colonialism. This would be to ignore the considerable internal political conflicts that have had constant negative impacts on unity and development. Yet, the devastating role of colonial power and arbitrary formula of the state in undermining community cannot be denied.

Finally, taking into consideration the destructive role of the colonial era and its disastrous consequences in weakening the *ummatic* system, it must be emphasized that Muslim governments themselves should also bear responsibility for continuing this status to the present time. The current unjust situation cannot be justified by holding the colonial powers responsible for every subsequent failure.

### Nation-state versus Umma: shedding light on the paradox area

Political entities throughout history have been varied. There were ancient kingdoms and empires that extended their authority over vast geographical regions. There were small political groupings such as tribes and clans. The modern nation-state was a unique political phenomenon due to its obsessive tendency towards nationalism, sovereignty, and territoriality. This tendency intensified in the colonial period, where the nation-state became a force against the development of a cohesive international community just as it stands against an attachment to the *umma* as a global community of Muslims (Evans and Newnham 1998, 27). In this regard, we can confine consideration of the contradiction between the modern nation-state and the *umma* to three fields—nationalism, territoriality, and sovereignty. These are the most relevant to making sense of the refugee crisis from an ethical perspective.

1. ***Nationalism***

Nationalism is one of the collateral contours of the modern nation-state^.^^17^ In his book, *The Impossible State*, Hallaq (2013, 7) contends that nationalism is an unprecedented phenomenon and uniquely constitutive of the modern state.^18^ Giddens (1985, 120) adds:

Both the nation and nationalism are distinctive properties of modern states and in the context of their original emergence as well as elsewhere there is more than a fortuitous connection between them.

The model of the nation-state was indeed constructed in Europe, with the Treaty of Westphalia (1648), as a solution to the bitter religious conflict between Catholics and Protestants. This religious feud led the Reformists to seek an alternative identity that could replace the religious identity that tore Europe apart. The national identity was chosen as their alternative and a savior.

The Protestant Reformation helped shatter the religious unity of Europe, and it was linked with the emergence of nation-states with their own boundaries, legislatures, jurisdiction—and therefore laws. It was a time of growing national consciousness. Vernaculars began to be used in universities, replacing Latin. There was a development of interest in national rather than Roman-based law. In Europe, legal nationalism eventually took the form of written national law codes. (Buzzard 1999, 68)

Gradually, nationalism intensified, differing in varying degrees from one country to another and crystallized in the idea of “nation above everything”,^19^ a motif of both World Wars. As such, some slogans nowadays like “America first”, “India First,” or other such rhetoric have become a rallying cry for a divisive politics of communities and international relations.^20^ The former Brazilian general Golbery, one of the most influential nationalist thinkers, stated:

To be nationalist is to be always ready to give up any doctrine, any theory, any ideology, feelings, passions, ideals, and values, as soon as they appear [to be] incompatible with the supreme loyalty, which is due to the nation above everything else. (in Wink 1998, 58)

In this sense, nationalism constitutes a threat to the *umma* as it is a source of division instead of cohesion. “Nationalism increasingly moves towards a form of “nativism,” which tends to extol local traditions and particularities in a chauvinistic fashion” (Tibi 1975, 25). “In fact, and in practice, state, nation, nationalism, and ethnic identity often become co-mingled in what is a heady and sometimes explosive mixture” (Pierson 2004, 48).

Indeed, the much more damaging aspect of nationalism as Michael Howard has suggested is that,

Nationalism was almost invariably characterized by militarism. Self-identification as a nation implies almost by definition alienation from other communities and the most memorable incidents in the group’s memory consisted in conflict with and triumph over other communities. (Howard 1978, 9)

With the rise of nation-states in the Muslim world, the priority was given to national identity instead of Muslim identity. Hence, the consciousness of Muslims as “one community” or *umma* began to wane. With the beginning of the twentieth century, this new identity was fueled by colonial powers as well as by the feeling of resentment that was triggered by Turkish nationalist movements. Those Turkish nationalists who were inspired by the Nationalist movements in the West sought “to inculcate the non-Turkish-speakers with Turkish nationalism by force” (Tibi 1975, 87). Consequently, many members of the *umma*, as a spontaneous reaction, began to identify themselves within a nationalist framework, such as Egyptian, Syrian, Iraqi, Indian, and Bangladeshi (Chatterjee 1993, 3–5). In his entry on Muslim Identity in the *Encyclopedia of Islam and Muslim World*, Peterson contended: “by the opening of the twentieth century, however, nationalistic movements in and about the Ottoman empire had destroyed more than the idea of political unity among Muslims, Christians, and others” (Peterson, 2003, 341).

Apart from the dire consequences that arose with nationalist movements, there were useful functions that resistance movements in the 1950s–1960s. In the Muslim World, the use of nationalism in that period was regarded by resistance movements as a part of the anti-colonial struggle and the war for independence in the Global South (Chatterjee 1993, 3–5).

However, nationalism can turn into ethnic politics, resulting in national conflicts that precipitate refugee and displacement crises. The attempt to impose cultural homogeneity on all the minority groups that exist within a country has resulted in some of the greatest calamities on human society (genocide, civil war, ethnic cleansing, persecution). These conflicts arise in large part due to the principle of creating one unified nation-state by “force”. As Anderson posits: “What makes the shrunken imaginings of recent history (scarily more than 2 centuries) generate such colossal sacrifices?” (Anderson 2006, 7). Moreover, when it comes to any influx or any threatened influx of migrants or refugees, it is seen as a major threat to national and ethnic foundations of the state (Geddens and Scholten 2016, 147–148).

2) ***Territoriality***

One of the main features that distinguish the modern nation-state from earlier political forms is its geopolitical character. It occupies specific territory and falls within demarcated and internationally recognized borders (Pierson 2004, 10). It would be a mistake to think that the borders in the traditional states such as the Roman Empire or China were akin to the borders in the realm of the modern nation-state realm today. As Giddens contends “In neither Rome nor China did the walls correspond to the limits of “national sovereignty” in the sense in which that term is applied today” (Giddens 1985, 51).

Borders in the modern nation-state are not mere geographical lines on a map. They are defensive militaristic frontiers against any potential invasion or influx from neighbouring or other states. Borders also work as “symbolic distinctions that crucially set apart a collective self from others” (Deleixhe et al. 2019). Therefore, another challenge posed by the modern nation-state against the *umma* is its territorial tendency, which promotes segregation between *umma* members and restricts their freedom of movement.

What is even more important to notice is that this territoriality enforces an emergence of “securocratic”^21^ borders, which aims to securitize every single movement that crosses. The problem of these securocratic policies for state borders is that they legitimize drone warfare and the everyday use of surveillance technologies against the so-called “threat,” that comprises a wide spectrum of undesirables ranging from drug dealers and terrorists to asylum seekers and undocumented immigrants (Halper, in Deutsch 2019, 3). Morally, governments have explained it as a necessity to practice the “least possible evil” if it prevents a “greater evil” from happening. Thus, it is “dangerous” where refugees and asylum-seekers are looked upon in the same way as terrorists and drug traffickers.

Although pre-modern empires, including the Islamic empire, were politically very extensive and powerful, their territorial limits were ill-defined frontiers rather than clear identifiable borders (Pierson 2004, 9). Borders of the state are neither neutral nor natural but rather a political instrument that is utilized by the state to confine people and delimit them into “citizens.” This means that citizenship determines the identity and the rights of persons and whether they have the right to move from one country to another. The general assumption is that borders exist to maintain security for the state and the social contract. However, strict borders are also an ethical–political practice, facilitating the freedom of movement for certain society members and discriminating against others based on the “value” of their citizenship (Vaughan-Williams 2009, 1–15). One serious consequence of these artificial border politics is the prevention of adequate responses to humanitarian issues and refugee crises. And it is precisely why the 1951 UN Refugee Convention (Article 31) was developed to provide protection for unauthorized entry of asylum-seekers seeking safety across state borders. The case of Rohingya refugees stuck in a small strip of land along the border between Bangladesh and Myanmar called “no man’s land” (whereby both countries evade the responsibility of their safety) is one example of a tragic crisis created by the territoriality of the nation-state (Ethirajan 2018). As some scholars argue, national borders and boundaries could be in fundamental contradiction with Islamic values, due to the Qurʾanic idea that human beings are trustees of the land and not its owners. The Universal Islamic Declaration of Rights (1981) states: “In view of the fact that the world of Islam is veritably *Ummah Islamia*, every Muslim shall have the right to freely move in and out of any Muslim Country” (ʿAbdul-Raḥīm 2005, 174).

3) ***Sovereignty***

Sovereignty marks one of the core components of the modern nation-state. Epistemologically, sovereignty, as defined by Hinsley (1986, 26), is “the idea that there is a final and absolute authority in the political community and that no final and absolute authority exists elsewhere.” Despite the ambiguity around who represents this sovereignty, whether the government or people, the essence of sovereignty rests within the limits of the state jurisdiction. In this context, “no other actor may gainsay the will of the sovereign state” (Pierson 2004, 12). Moreover, this sovereignty is acknowledged and respected by other nation-states and international organizations (Adiong 2013, 15). Hence, the state becomes “the author of its own laws” (Morris, 2004, 198).

In this respect, the sovereignty of the modern nation-state system stands in opposition to the concept of the *umma*, which devotes the absolute authority to divine guidance instead of the people or a state. Muslims believe that Islam provides the *umma* with a complete legal and moral system of life, not only in the individual domain but also in the social and political sphere. From an Islamic theological point of view, the *sharīa* is more authoritative than the state and all sovereignty belongs to the divine. Thus, an Islamic government is nomocentric (law-based), where “law precedes the state, which exists for the sole purpose of maintaining and enforcing the law” (Lambton 1981, xv; Laknāwī 2002). On the contrary, within the modern nation-state, sovereignty means that the state has the absolute power to decide its laws and legislative system without any restrictions (Laknāwī 2002). The following passage by Hallaq (2013) has nicely captured this meaning:

Whereas the nation-state is the end of all ends, knows only itself, and therefore is metaphysically the ultimate foundation of sovereign will. The Community (*umma*) and its individual members are a means to a greater end. This implies that the Community (*umma*) itself neither possesses sovereignty nor does it have—in the sense the modern state has—an autonomous political or legal will, since the sovereign is God and God alone. Of course, the Community as a whole, and as represented by its chief jurists, does have the power of decision, this being the crux of the doctrine of consensus. But this power is an interpretive one, bounded by general moral principles that transcend the Community’s control. (Hallaq 2013, 42)

Here, there is an issue related to sovereignty that needs to be re-examined, namely as a democratic tool reflecting the people’s will in a modern nation-state. Realistically, this is not consistent because “even when everyone knows that a regime is unrepresentative and even oppressive, it is still deemed to speak legitimately on behalf of its citizen/nation” (Hallaq 2013, 28).^22^ When it comes to the impact of the sovereignty of a modern nation-state on the idea of an *umma*, it turns out that sovereignty allows the state to deal with humanitarian crises of other states as it wishes. Religious obligations and values are to have little influence in the political decision of acceptance or rejection of refugees (or any “foreigner”). More often, perhaps, is the appeal to the alleged “national interest” which is deliberately ambiguous. Even states that espouse human rights, the identification of national interest can lead to “jingoistic” arguments and very destructive consequences (Cole and Kandiyoti 2002, 198). The idea of unbridled sovereignty destroys the *umma* idea that obligates solidarity and fraternity between members of the *umma*, not the other way around.

To sum up, these aspects of contradiction between the *umma* and the nation-state resulting from the coercive and arbitrary imposition of a nation-state model cannot be reconciled without restructuring the nation-state, particularly the Muslim nation-state, on principles that transcend the modern tenets of nationalism, territorialism, and sovereignty. Thus, it is, that *ummatic*, humanitarian and ethical principles of solidarity, regardless of national, cultural, and religious characteristics, should be the defining principles of the nation-state. In a world where asylum-seekers are not being given the protection they require, the “nation-state” formulated by seventeenth century European thinkers and arbitrarily imposed by imperial powers has become increasingly unwilling to avail them of their protection needs. Muslim nation-states can be rationally and morally effective in addressing refugee problems. They could provide more stability both nationally and internationally, encouraging economic integration and promoting human rights in a framework that is compatible with the *umma*—and without compromising citizenship rights. States of the European Union, for example, managed to preserve their sense of national identities, languages, and ethnicities as they proceeded to unify over political, security, economic, and monetary issues (Wilets 1999 228–229).^23^

### Refuge, migration ethics, and umma: a study of inseparable relation

It is important to note that refugee rights in Islam are closely associated with the sentiment of attachment to the *umma* in its conceptualization as a global Muslim community. For an effective application of Islamic ethics regarding asylum, there needs to be first and foremost an *ummatic* feeling along two dimensions. First, there must be a brotherhood/sisterhood-in-faith, which extends across borders; and second, that the *umma* bears a religious and ethical responsibility towards the persecuted and needy, whether Muslims or non-Muslims. With regard to the first dimension, the Qurʾan links all Muslims with a deep-seated tie—the tie of faith. The Qurʾan says, “Surely all believers are brothers” (Q 49:10). This verse is one of the most important in relation to the rights of refugees. There is no brotherhood/sisterhood as long as a Muslim does not feel the suffering and worries of his/her refugee brothers/sisters and does his/her best to eliminate or relieve this suffering. This undoubtedly requires sheltering refugees in Muslim countries in a just manner. The Prophet said, “The believers are like a single structure, each part supporting the other” (Bukhārī 1933, 3/129). The centrality of brotherly love in Islam may be best expressed in the famous *ḥadīth*: “None of you truly believes until he loves for his brother what he loves for himself” (Bukhārī 1933, vol. 1,13). This *ḥadīth* goes to the heart of the *sūra* (chapter), which emphasizes a bond of faith transcending those of kinship and suggesting that one should no longer make alliances based merely upon tribal bonds (Nasr 2017, 2286)—to which one might add national allegiances.

Likewise, the Prophet expressed clearly this meaning of attachment to the *umma*, saying, “The believers, in their mutual love, mercy, and compassion are like one body: if one organ complained, the rest of the body develops a fever” (Bukhārī 1933, vol.8, 10). This *ummatic* feeling is expressed in many authentic hadiths. For example, “The Muslim is the brother of a Muslim. He does not do him injustice, nor does he abandon him” (Bukhārī 1933, vol. 3, 128).

In this sense, social development in the Arabian Peninsula has been attributed to the *ummatic* system that went beyond tribalism in order to establish trans-boundary ties formed by Islamic ethical values. According to Agha:

The collectivist *weltanschauung* of the *ummatic* value system was a landmark development in Arabia, as it succeeded in shifting the allegiances of fiercely tribal peoples to a set of common religious beliefs that would mutually benefit communities beyond the boundaries of individual tribes. Moreover, the *ummatic* concept had greatly tempered the excesses of the harsh tribal code, characterizing centuries-old traditions and mores of the Arab desert-dwellers. (Agha 2008, 37)

With reference to the second dimension, the ethical responsibility upon the *umma* towards non-Muslim refugees, it is useful to recall the practices of the Prophet Muhammed and Muslims in the *hijra* (migration) to understand how much refuge ethics are inter-linked to the *umma*. Within Islamic history, the *hijra* is not a mere historical event, but rather a tipping point that had featured in the history of the *umma*. There were two major migrations (hijras) in the Prophet’s time. The first one was at the advent of Islam when the early Muslims faced different types of persecution and torture from the pagans in Mecca. The continuous abuse and mistreatment forced the Apostle to invite his followers to emigrate from Mecca to Abyssinia (Ethiopia) which was ruled by a Christian king, Negus, well known for being just and protective of his people. King Negus welcomed the Muslim refugees and gave them sanctuary, security, and freedom of worship. Ibn ʿAbdul-Barr (1982, 52) stated: “When those landed in Abyssinia, they felt safe with their faith and stayed in the best homes and enjoyed the best protection.” The king refused to extradite them and said to the Quraysh’s envoys: “Even if you give a mountain of gold, I won’t surrender them to you” (Ibn Qayyim al-Jawzīyya 1994, 26). This historical incident, which has been cherished by Muslim scholars, was the first *hijra* in Islam, and it forms a well-established maxim of refuge ethics in Islam, invoking the principle of *non-refoulement*, one of the most important provisions in the 1951 UN Refugee Convention. It has always been used as an indicator, or model, that the protection of those fleeing persecution and oppression is an act of justice from an Islamic perspective.

The second and most notable *hijra* was the flight of the Prophet from Mecca to Medina to save his followers from persecution and to put his faith’s commandments into practice; that is, to escape from a place where one faces oppression and cannot practice his/her faith (Schmiedel and Smith 2017, 90–91).

However, an important consideration in Islamic ethics of migration that is relevant to the contemporary debates about distinguishing between refugees and economic migrants may be located in the Qurʾan that records: “And if anyone of the polytheists seeks your protection, then grant him protection so that he may hear the words of Allah. Then deliver him to his place of safety” (9:6). This verse clearly requests Muslims to extend protection to those who are in need and who ask for protection, even if they are non-Muslims and even though they may be enemies of Islam. No Muslim should refuse to accept any request for asylum and refuge. Here also is the principle of *non-refoulement*, articulated in the notion of sending the asylum-seeker to a place of safety. Most interestingly, there is no requirement for the asylum-seeker to justify why protection is being sought. In theory, the motivation for seeking asylum is neither requested nor considered, so there is no distinction between a refugee and a migrant, for there are no conditions attached to the obligation to provide protection for those who ask.^24^

One often-cited example of fulfilling this ethical duty is the haven that was given by Muslims, as *umma*, to the Jews when they faced inquisition and persecution in Europe during the end of the fifteenth century:

The Ottoman Empire proved a haven for the Jews of Mediterranean Europe and the Middle East. Ashkenazis from Germany, France, and Hungary; Italian Jews from Sicily; and Sephardic Jews from Spain and Portugal settled in Ottoman domains. Mehmed the Conqueror invited Jews from Anatolia, Salonica, and Edirne to move to Istanbul. (Esposito 1999, 386)

Garcia-Arenal eproofing@springernature.com added: “Jews who were expelled from the Iberian Peninsula, Calvinists from Hungary and Transylvania, Silesian Protestants and Russian Cossacks. All these sought refuge in Ottoman territory or requested assistance from the Sublime Porte in their struggles to resist Catholic or Orthodox persecution” (García-Arenal 2011, 603). A notable example from more recent history are those Jewish refugees who were granted asylum in Istanbul during World War II as Turkey accepted Jews from Germany, giving them immediate citizenship status (Öztürk 2009, 77).

In short, this link between the *umma* concept and refugee rights inevitably imposes itself in the Islamic legal structure, without which the system would be incomplete. Hence, the question that will be discussed in the next section of this paper is whether the *ummatic* system still exists today in Muslim-majority countries in relation to refugees and Muslim refugees or whether it has ceased to exist due to the dominance and counter-interests of the modern nation-state? And is it desirable to reform these policies in the light of the *umma* concept to broaden the humanitarian options for refugees?

### Refugee policies in Muslim-majority countries

Many commentators suggest that there are, in general, no effective policies in place that can provide refugees with adequate protection and commensurate with the standards of the international law. In a report on asylum in the Mashrek,^25^ the researchers concluded that:

Migrants and refugees in the Mashrek region suffer from many violations of their rights. They are at risk of unlawful deportation, ill treatment and exploitation, and may face great difficulties in claiming their most basic rights, such as healthcare, education, housing, and work. Several different factors limit migrants’ and refugees’ access to their rights. (Hilal & Samy, 2008, 10)

The status of refugee and asylum polices has been also examined by Zaiotti (2006) by raising state-centric questions concerning the levels of implementation of international law, ratification of conventions, and the passing of local regulations. He concluded that:

Apart from the Palestinian case […] No formal provision regulating the status of refugees has been devised, and few countries in the region have acceded to the main legal instruments defining the international refugee regime. Policies towards these individuals therefore have been formulated on an ad hoc basis. As a result, refugees have enjoyed few guarantees and minimal protection. (Zaiotti, 2006, 334)

To use one refugee lawyer’s traumatic phrase, “there is basically no refugee policy in the Middle East”. There are only refugee problems and—at best—occasionally some ad hoc and discretionary steps taken to alleviate suffering for short periods of time (Kagan, 2011, 8). Yet, it was acknowledged that “there are some systems in place to receive people fleeing persecution; some refugees are able to find shelter, though many people are likely to fall through the cracks and the amount of protection available is certainly quite limited” (ibid, 9).

Here, Zaman argues that refugees in Muslim countries are not being integrated due to the absence or weakness of the sentiments of belonging to the *umma*. The response to the refugee influx “has been characterized by ambivalence, ambiguity, and even paradox on the part of majority Muslim states”. It is in the encounter between settled resident populations and the arrival of the newly displaced (Zaman 2020, 3).

Most Arab governments are likely to acquiesce to the presence of refugees as long as their responsibility is visibly assigned to an international body or other third party (Kagan, 2011). However, all efforts to integrate refugees are likely to be blocked by policies that prevent work permits, nationality, permanent residency, or by the silent machinations of the modern nation-state (ibid). Without shifting responsibility, these governments would be forced to face the contradiction between the presence of Muslim refugees and their non-integration. They may resolve this contradiction the Egyptian way, where there is a systematic “non-enforcement of laws on the books, leaving refugees in a fragile state outside the rule of law”. A state might try to regularize the status of refugees by creating an alternative third-party sponsor. Or the state might respond by simply expelling them (Kagan, 2011, 13–14). The application of the *umma* is a call for proper integration with dignity.

### Refugee policies of Turkey, Jordan, and Bangladesh

These three countries reveal a slightly different picture to that depicted above. From our cursory observations, there is no official *umma* refugee policy in Turkey, Jordan, or Bangladesh, but some nuances are required. Turkey, Jordan, and Lebanon have accepted around 4 million Syrian refugees since 2011.

Turkey has ratified the 1951 Refugee Convention but with the geographical limitations from not signing the 1967 Protocol. Neither Jordan nor Bangladesh have signed the Refugee Convention, but Jordan has made its own arrangements with UNHCR, yet all three countries have allowed for a large number of refugees to enter their countries. Currently, Turkey hosts the largest number of refugees in the world. The largest refugee group in Turkey are Syrians (3.6 million), followed by Kurds (1 million), then Afghan nationals (182,000 registered and and approximately 120,000 not registered) (Augustova and Hakimi 2021). Because of Turkey’s geographical limitations, Afghans have not been able to seek asylum, or the temporary protection afforded to Syrian refugees. Officially, Afghans are thus considered to only be transiting in Turkey. They can seek International Protection status but to be resettled to a third state (see ICMPD 2021). However, because of negligible resettlement opportunities registered Afghan refugees have been waiting 5–10 years, “with only one year of access to public medical care and no access to work permits or financial support from the government” (ibid: 10). Questioning whether Turkey remains a host country for Afghans, more recently, “Turkey has shifted towards mass deportations and push-backs of Afghans, preventing them from seeking protection within its borders” (ibid: vi).

Up until, these very recent restrictions and deportations; however, Turkey has been a relatively generous host to the largest population of Muslim refugees in the world. To what extent were these because of political decisions, or because they were neighboring area countries and unable to ignore the humanitarian crises? This is not something easily answered. It is noteworthy that in May 2013 following a car bombing in Hatay, the Turkish president Recep Tayyip Erdoğan clearly invoked *ummatic* symbolism for accommodating the displaced Syrians and perhaps for regional politics:

My siblings in Reyhanli should serve as *anṣār* to the *muhājirūn*^26^ who fled from the brutality of al-Assad. They should fulfill the same duty. They should also open their homes exactly like it happened at the time [of the Prophet]. (*Hurriyet Daily News*, May 24, 2013; cited in Zaman, 2020, 52)

However, the Turkish Minister of Work and Social Security, Faruk Ҫelik adopted a different stance in relation to the Syrians’ right to work. He stated: “It would be unfair to take away their [local Turkish] jobs and give them to refugees” (*Reuters*, 07 August 2015). This stance was contrasted with the position of Fatma Şahin, the Mayor of Gaziantep from the AKP, who declared:

People took refuge in our city to protect their lives and their families after the civil war in Syria. It wasn’t their choice but an existential necessity. They took refuge with their neighbours they saw as a safe port. Being neighbours is a sacred relationship according to our beliefs. (cited in Zaman, 2020, 51-52)

Notably also, Turkey supported Rohingya refugees outside of its territory. Turkish Ambassador to Bangladesh, Mustafa Osman Turan, stated that “Turkey’s contributions to the welfare of Rohingya Muslims in Bangladesh’s Cox’s Bazar camps exceed $60 million, besides the aid which is dispersed through the UN agencies”.^27^

Thus, there is an oscillating between *ummatic* political motivations of acceptance and more hostile or cautious attitudes “standing at the threshold of a sedentarist world created by the nation-state” (Zaman, 2020, 50). Yet, prior to the Syrian crisis, it is well known that thousands of Somali asylum seekers crossed the Gulf of Aden to Yemen and from there traversed Saudi Arabia, into Syria, and then to Turkey where they registered with UNHCR as refugees with hope to launch into Greece and Europe from there. This trajectory, which was fraught with traumas along the way, was made possible because they were only transiting in Saudi Arabia and, as Muslims, did not require a visa to Syria (Jureidini, 2011). Very recently, Greece has accused Turkey of “encouraging Somali migration through promotion campaigns and visa offers and then allowing the migrants to continue to Greece”,^28^ yet this route for Somalis has been active for many years.

The Kingdom of Jordan did not ratify the 1951 Refugee Convention but did enter into a Memorandum of Understanding (MOU) with UNHCR. The memorandum formalizes the relationship between the Kingdom and the refugees and serves as a source of international law in describing the real refugee system on the ground. The Jordan MOU describes the presence of refugees as a “sojourn and gives force to the transit country concept by imposing strict time limits on refugee’s residence, 6 months only”.^29^ Taking into consideration the huge number of Palestinians refugees (from 1948, 1967, and 1973) who have been accommodated and provided full integration and granted Jordanian nationality may explain this reticent and harsh position towards accommodating other refugees. Again, looking at the political decision may complete the picture. In his speech to the “Supporting Syria and the Region Conference,” King Abdullah II emphasized the breadth of Jordan’s refugee assistance, but without invoking the *umma*:

Today, one of every five people living in our Kingdom is a Syrian refugee. It is as if the UK had to absorb the entire population of Belgium. Hosting Syrian refugees consumes more than one quarter of Jordan’s national budget. The USA and EU, whose combined economies are almost a thousand times bigger than Jordans, are struggling to handle about one million refugees: our small country, alone, has taken in almost 1.3 million already. And this is in addition to all the others whom Jordan shelters from the Gulf wars, the Palestinian-Israeli conflict, Libya, Yemen, as well as Christian minorities fleeing persecution.^30^

Note that Jordan accommodates Afghan as well and Rohingya refugees even though Afghanistan and Myanmar are not neighboring countries to Jordan.^31^

For Bangladesh, with almost 1 million Rohingya refugees in Cox’s Bazar, “it is a big challenge as it is creating security problems for locals and disrupting the social balance.”^32^ Typical of neighboring countries, the government did not prevent them from entering the country, but it also does not allow them to work and build schools or hospitals and they are not allowed to move out of the camps to live in other cities of Bangladesh—i.e., to integrate as one of the durable solutions for refugees. Since the Burmese military coup in 1962 and their subsequent loss of citizenship status in 1982, hundreds of thousands of persecuted Rohingya have been fleeing Myanmar into Cox’s Bazar in Bangladesh. Most were repatriated back to Myanmar/Burma but many remaining in exile (Minority Rights Group International, 2019). However, 2017 was the largest single movement of 745,000 by the end of 2017 fleeing the most unimaginable horror that was captured on television around the world. The Bangladesh consul in Qatar stated that they did receive them “as Muslims,” but they would do the same with Christian or Jewish refugees as an ethical and international obligation. The statement however does indicate an Islamic and thus *ummatic* preferential recognition towards the three monotheistic religions.

Nevertheless, it was always an uneasy acceptance by Bangladesh. By October 2018, agreements were being reached between the two countries for the Rohingya to return, but the safety and citizenship had not been secured and the people were unwilling to go. By the end of February 2019, Bangladesh announced the closing of the border to prevent further crossings (ibid). At the end of August 2020, Human Rights Watch (HRW) reported that after 3 years, Myanmar was still not safe for Rohingya refugees to return and that “Rohingya refugees in Bangladesh have faced tightened restrictions on rights to information, movement, and access to education and health and have been unlawfully killed by Bangladeshi security forces” (Human Rights Watch 2020). Even Malaysia has treated them as pariahs.

Some have been stranded at sea for weeks or months, with hundreds feared dead on boats that disappeared after Malaysia and Thailand illegally pushed the back using the COVID-19 pandemic as justification. Malaysia has detained Rohingya refugees, denied them access to the UN refugee agency, and prosecuted some for illegal entry. Despite pledges, the Bangladesh government has yet to allow Un officials to assist the over 300 Rohingya refugees rescued at sea and currently detained on the insecure silt island of Bhasan Char. (Human Rights Watch 2020, 2)

While the Consuls of Bangladesh, Turkey and Jordan stated that they have welcomed the Syrian, Rohingya, or Palestinian refugees because they are part of the Muslim *umma*, they could not point towards any specific policy that explicitly gives preference to, or contains an obligation for, them to accept Muslim refugees.

In general, it becomes clear that perhaps most Muslim-majority countries have adopted a “non-integrative policy approach,” which means that they may offer refuge in their territory, but only temporarily and do not grant them many of their rights as refugees.^33^ They do not offer a durable solution. As mentioned above, GCC countries have been accused of not taking in any refugees, whereas there have been studies that prove that they indeed took in a limited number of refugees, but in the name of “guests” or more accurate expression “employees” under the sponsorship (*Kafala*) system, which means that there is no chance of granting them refugee status and placing them under the conditions of international protection. Nevertheless, GCC country refugee programs are more inclined towards supporting refugees by being one of the top aid-contributors to humanitarian operations in refugee camps for instance. In the case of the Syrian crisis, GCC countries have contributed a substantial amount of money, a total of US$910.3 million in 2013. “US$690.8 million was given by four Gulf States [Kuwait US$325 million, Saudi Arabia US$213 million, United Arab Emirates US$85.7 million, and Qatar US$66.2 million] and US$219.5 million by Gulf-based NGOs and state-organized humanitarian institutions.”^34^ This financial generosity, however, has prompted the criticism that it maintains a distance from the suffering and is therefore more like a “politics of pity,” rather than a “politics of compassion” (Hanafi, 2017).

### OIC and the incorporating umma concept into refuge policies

We raised at the inception of this paper an important question regarding the potential possibility of reforming refugee policies and refugee status through an inspiring *umma* concept, which demands fraternity and solidarity. The idea of integration of the people of the *umma* as is a sentiment that may be mobilized to accommodate refugees on both political and humanitarian levels. Thus, there is a need for reviving this concept and applying it in reality. The idea is that integration should be in terms of legal, economic, social, and cultural dimensions with members of the host country that they are not marginalized but social cohesion and harmonious existence is fostered (UNHCR 2014).

As has been shown, the history of the Islamic *umma* as a paradigmatic concept has been checkered and is inconsistently used or invoked when it comes to Muslim-majority countries and their policies toward refugees. The nation state and its political-economic interests seem to hold sway in many circumstances, but not all. The study of Islamic ethics, like the study of any ethics, is always beset by a perennial mismatch between the principle and the practice.

A study issued by the United Nations High Commissioner for Refugees entitled “The Right to Asylum Between Islamic Law and International Refugee Law: A Comparative Study” (Abou El-Wafa, 2009) revealed that the customs followed in Islamic law 1400 years ago in sheltering people fleeing persecution have a greater impact on contemporary refugee law than any traditional source elsewhere. According to Antonio Guterres:

Islamic law, more than any other historical source, is the basis of the legal framework on which the UNHCR builds its activities aimed at assisting tens of millions of people fleeing their homes around the world.^35^

### UNHCR refugee zakat fund

The UNHCR has already adopted the Islamic charitable institution of *zakat*, one of the 5 pillars (obligations) of Islam. In 2019, it introduced the Refugee Zakat Fund, backed by Islamic scholars and fatwas to tap into Islamic charitable giving in the face of declining UN resources (see: https://zakat.unhcr.org/en/about-zakat).

The first Zakat Initiative was introduced by UNHCR as a pilot program in September 2016, partnering with the UAE non-profit Tabah Foundation (Cole 2021). In April 2019, the initiative was transformed into the Refugee Zakat Fund, to “help individuals and Islamic financial institutions to realize their social responsibility with global impact” (Cole 2021: 56). It was from the outset seen as a pragmatic collaboration between Muslim philanthropy unable to reach its constituency, and a UNHCR that was unable to meet the assistance needs of refugees and the displaced. With over 70 million forcibly displaced globally, 60% are from the Organization of Islamic Cooperation (OIC) countries (Kidwai and Zidani 2020: 45). The fund initially sought to address the needs of mainly displaced Muslims in states that were not signatories to the 1951 Refugee Convention, namely, Jordan, Lebanon, Bangladesh, Malaysia, and Iraq and the signatory states of Egypt, Yemen, and Mauritania.

This was not the first collaboration between Zakat and UNHCR. Many former Muslim contributions to refugees, including involvement of the OIC, are detailed in Muzaffar (2001) that shows the idea had been gestating for at least two decades. Briefly, for example, Kuwait’s Zakat House in the 1990s partnered with UNHCR, supporting UNHCR activities in Kuwait, Chechnya, and Syria (Muzaffar 2001: 259). And in Kuwait, Zakat House provides UNHCR’s Persons of Concern with 100 KD [US$330] every 3 months (Svobodová 2018). In 1999, UNHCR acknowledged the great potential of a proposal by Prince Hassan of Jordan to create an International Foundation for *Zakat* for the benefit of refugees (Muzaffar 2001: 262).

In UNHCRs pilot phase, 2016–2018, *Zakat* beneficiaries to refugees and IDPs numbered 34,440. In its first year (2019), UNHCR Refugee Zakat Fund assisted over 1 million beneficiaries, distributing US$43.165 million. More than 90% of this came from Muslim foundations and charities, primarily the GCC, who themselves chose the destination countries for their contributions (Kidwai and Zidani 2020: 52). In 2020, the UNHCR Refugee Zakat Fund received US$61.5 million, 79% being *Zakat*. These funds reached over 1.6 million beneficiaries in Jordan, Lebanon, Iraq, Yemen, Egypt, Mauritania, Niger, Pakistan, India, and Bangladesh—a 59% increase from 2019 (UNHCR 2021). Thus, UNHCR came to be viewed as “a trusted, compliant, and effective distributor” (UNHCR 2022: 1).

In 2019, through his humanitarian foundation,

H.E Sheikh Thani Bin Abdullah Bin Thani Al-Thani made a landmark Zakat contribution of more than USD 35 million in support of Rohingya refugees in Bangladesh and displaced Yemenis through the fund. This was the largest contribution UNHCR had ever received from an individual donor and the first major Zakat contribution of its kind in the organization’s history. A total of approximately 875,000 Rohingya refugees and Yemeni IDPs, returnees, and members of the host community were supported through this generous contribution. In 2020, and for the second year in a row, Sheikh Thani Bin Abdullah Bin Thani Al-Thani Humanitarian Fund continued aiding hundreds of thousands of people through the largest individual contribution to UNHCR in the organization’s 70-year history. More than USD 43 million were directed to support the most vulnerable internally displaced and host community families in Yemen, as well as refugees in Lebanon, Bangladesh, Pakistan, and Chad. (UNHCR 2021: 26)

Without reliable data, Stirk gives a wide estimate of between US$200 billion to US$1 trillion is spent annually “in “mandatory” alms and voluntary charity across the Muslim world” (Stirk 2015: 6). Alternatively, Ali al-Qaradaghi, the Secretary General of the International Union of Muslim Scholars, estimates $100 billion annually as the value of Zakat raised from companies, banks, and individuals in the GCC countries. By contrast, “Indonesia, Malaysia, Qatar, Saudi Arabia, and Yemen, which account for 17% of the world’s Muslim population, collect at least $5.7 billion annually” (Stirk in Derbali, 2021: 2). Interestingly, Cole points out, “UNHCR has also been trying to harness individual donations, particularly from “tech-centric Muslim millennials” in the Gulf States. In 2019, nearly 60% of all digital donations to the Refugee Zakat Fund came from individuals based in the UAE and Saudi Arabia” (Cole 2021: 57).

From an Islamic perspective, it is important to note that donating and giving *Zakat* (and other forms of charitable giving) is not only a religious act, but its function is also to serve social solidarity among Muslims as in the *Umm*a. For example, Kasim and Towadi use the case of Indonesian assistance to the Rohingya to argue that the idea of *Zakat* to finance Muslim refugees was to “strengthen obligations to protect the Rohingya refugees as solidarity among fellow Muslims” (Kasim and Towadi 2018: 23). Similarly, Derbali suggests that “the international *Zakat* fund aims to promote concepts such as justice and social equity as well as solidarity and mutual aid” (Derbali 2021: 4.1).

This is reiterated in the policy of Kuwait’s Zakat House that “aims to strengthen the solidarity and interdependence of all Muslim people who actually constitute one *Umma* despite the remoteness of their countries of residence” (Abu-Ghodda, in Muzaffar 2001: 260). Emphasizing the practicalities of using the aid infrastructure of UNHCR, it is argued that “Faith-based organizations should lead the way while capitalizing on strategic and sustainable partnerships with multi-lateral organizations based on an effective and impact-oriented approach …[and] … build a sense of solidarity with their host Muslim brothers and sisters” (Kidwai & Zidani 2020: 49). The UNHCR Refugee Zakat Fund can thus be located firmly within the Muslim ethical framework of the *Umma.*

Kidwai and Zidani (2020) also lament that “in many countries Zakat is managed with only a concern for absolute poverty relief, rather than social empowerment of the whole community” (Kidwai & Zidani 2020: 46). The gathering and dissemination of financial aid through Zakat is for them a scriptural goal “to strengthen spiritually through economic empowerment of Muslim communities” (ibid: 47). Thus, the use of Zakat is not merely a response to immediate urgent humanitarian needs, but also long-term community development towards self-sufficiency, which is better facilitated by cash donations. “If Zakat was only about poverty and the poor”, they argue, “why would there be a separation between voluntary charity [*Sadaqah*] and obligatory Zakat?” (ibid: 48). This is consistent with the observation that “eighty-nine percent of humanitarian aid goes to places that have required humanitarian funding for more than 3 years, and 66% of humanitarian aid is spent in places that have needed it for 8 years or more” (Center for Global Development 2015: 7).

UNHCR had to agree to uphold three key principles as laid out in the 5 main *fatwas* that have modified the organization’s normal operations.

1. Recipients must be one of the eight eligibility categories as stated in the Quran (9:60) (most coming under five categories: the poor, the needy, the indebted, stranded travelers, and those in bondage).
2. All funds must be distributed without deductions for administration overheads (normally 7%).^36^
3. Donations should be in cash (bank transfers or hard currency) whenever feasible, as a more dignified means of support, facilitating better monitoring. (Cole 2021)

These arrangements are “donor-centric” with the added proviso that “the destination of the funds is decided by the Zakat donor/partner at the onset of partnership” (UNHCR 2022: (1) Further, aid dissemination must be in accordance with the 8 categories of beneficiaries to be targeted by Zakat—according to the Quranic Verse 9:60 of Surah At-Tawbah—namely: (1) the poor (low-income or indigent), (2) the needy (someone in difficulty lacking resources), (3) those employed to collect Zakat (administrators of Zakat), (4) those inclined to Islam (converts and friends of Muslims), (5) those in bondage (slaves, captives, those enslaved by uncertain circumstances or persecutors), (6) the insolvent (debt-ridden to recover things lost), (7) for the cause of Allah, and (8) the wayfarer in distress (stranded travelers, refugees). In reality, eligible beneficiaries that are most in need among refugees and internally displaced persons come under five of the eight categories of Zakat: Poor—*Al Foqara’*; Needy—*Al Masakeen*; Debtors—*Al Gharimun*; Wayfarers or Stranded Travelers (refugees)—*Abna’ Al Sabeel*; in Bondage—*Fil Riqab* (Kidwai and Zidani 2020: 50).

By 2021, UNHCR claimed to be “a trusted, compliant, and effective distributor harnessing the power of Zakat to transform the lives of refugees. The Fund is subject to rigorous governance, ensuring transparency at every step, from donation to distribution of Zakat funds to eligible families” (UNHCR 2022: 1).

It is worth noting that all cash received by the fund is distributed in cash (or consumables) to beneficiaries without any deduction for overheads. These are both requirements by the Zakat fatwas and justified by the preponderance of vulnerable families, “often headed by women alone, who often face cultural, legal, and childcare obstacles when looking for work” and resort to desperate survival strategies “such as pulling children out of school and child labour” (Kidawai and Zidani 2021: 51). Cash assistance ensures that the immediate needs of families are met according to their own priorities. This also means that thousands of refugees who are restricted from working in host countries avoid facing severe hardships, while being empowered to manage their own finances. It helps to improve social networks through enabling refugees to repay loans borrowed from family, neighbors, and landlords. Additionally, it benefits the local economy as refugees become direct customers at local shops and markets (UNHCR 2021).

Cash assistance, identified as the most appropriate for *Zakat* distribution, is at the heart of the *Zakat* distribution policy. Financial governance includes the following:

- Management and accountability: funds are kept in a dedicated interest-free bank account in Geneva.
- Traceability: all funds are dedicated to Zakat compliant cash assistance programs in identified countries with high needs.
- Transparency: UNHCR publishes annual Zakat reports, and partners are provided with customized reports. (UNHCR 2022: 2)

Humanitarian aid normally provides physical commodities such as food, shelter, water, tents, clothing, and medical help. The Center for Global Development (2015) argues that supply convoys with sacks of food are now being replaced by “an envelope of cash, a plastic card or an electronic money transfer to a mobile phone, with which they can buy food, pay rent and purchase what they need locally” (Center for Global Development 2015: 7). They argue that when possible, cash transfers should:

- a. Align the humanitarian system better with what people need, rather than what humanitarian organizations are mandated and equipped to provide
- b. Increase the transparency of humanitarian aid
- c. Increase accountability of humanitarian aid, both to affected populations and to the tax-paying public in donor countries.
- d. Reduce the costs of delivering humanitarian aid and so make limited budgets go further.
- e. Support local markets, jobs, and incomes of local producers.
- f. Increase support for humanitarian aid from local populations.
- g. Increase the speed and flexibility of humanitarian response.
- h. Increase financial inclusion by linking people with payment systems.
- i. Most importantly, provide affected populations with choice and more control over their own lives. (Center for Global Development 2015: 8)

By 2020, Islamic finance banks provided automatic accounts deductions for *Zakat*, while blockchain technology now enables *Zakat* tracking from donor to recipient (Kidwai and Zidani 2020: 47). By the end of 2020, UNHCR launched the first official Zakat mobile app, “GiveZakat”, the most prominent feature “being real-time reporting on Zakat funds received online, in addition to a tracking tab allowing users to trace their Zakat donations until they reach beneficiary families” (Khalid Khalifa in UNHCR 2021: 4). At the beginning of September 2021, UNHCR announced the expansion of the Refugee Zakat Fund to displaced families in Afghanistan, through the GiveZakat App or the Refugee Zakat Fund website (UNHCR 2021).

Thus, the increasingly universalized outreach using the latest technology and United Nations infrastructure for the accumulation of obligatory Muslim charity funds and their global dissemination, progressively speaks to the practice of *Umma*, if the original socio-religious objectives are manifested. This leads to the issue of religious exclusivity and discrimination.

Cole states, “most *fatwas* specify that these recipients should be Muslims. As 60% of displaced people worldwide are currently eligible to receive Zakat; however, this is unlikely to require any change in UNHCR’s activities for the foreseeable future” (Cole 2021: 58). The religious imperative of *Zakat* may be seen as part of the *umma* in the sense of community commitment, but here specifically targeting the poor and needy (Derbali 2021). At the same time, it has been noted: “In effect, the Qur’anic recognition of such rights of the less fortunate demands a corresponding duty of the Muslim society collectively to guarantee a minimum standard of living for both Muslims and non-Muslims in the Islamic state, including refugees” (Muzaffar, 2001: 255).

Importantly, however, it is the religiosity of both donation and acceptance of *Zakat* that suggests the need for discrimination.

While collection and payment of Zakat are an act of faith, it’s best led firstly by agencies that are able to affirm the faith and build structures to ensure Zakat funds are managed with due regard…. When this is done with a sound social welfare strategy founded upon scripture, this could help enhance impact, reduce cost and improve reach. (Kidwai and Zidani 2020: 49)

It would seem at this historical juncture that UNHCR acceptance of a Muslim exclusivity in *Zakat* donation and dissemination is a pragmatic one. However, it may be seen as violating the principle of non-discrimination and debate on this issue will surely ensue. Yet, there are also differences of opinion within the community of Muslim scholars. The large majority agree that Zakat is a redistribution mechanism between rich Muslims and poor Muslims. Those who are not eligible recipients are (1) the rich, (2) those capable of working and earning, (3) spiritual devotees who refuse to work, (4) committed unbelievers and renouncers of Islam, (5) other non-Muslims, and (6) the children, parents, and wives of male Zakat donors (Zakat Foundation 2022). With a few exceptions, generally speaking:

While the majority opinion is that Zakat cannot be given to non-Muslims, there is a well-argued and supported minority opinion based on the Hanafi ruling, upheld by other eminent scholars, that Zakat Al-Fitr (the second kind of Zakat) can be given to the People of the Book (Jews and Christians, and those analogous to them). Based on this, eminent scholars, both classical and contemporary, hold that Zakat may be paid to the poor of the People of the Book, provided that (a) the prior right of the Muslim poor and needy to the Zakat wealth paid by the Muslim rich alone is not impaired or curtailed in any way by this and (b) that the poor of the People of the Book to whom this excess Zakat is paid do no harm to Muslims or to the religion of Islam. (Zakat Foundation 2022)

Some scholars hold that the very word “needy” (*miskîn*), as one of the eight eligible types prescribed by the Quran to receive Zakat, refers not to Muslims but to the People of the Book, though this is not the position of most scholars.

As has been observed, the UNHCR Zakat Fund reports do not address this issue and it is not clear from these reports whether Zakat recipients must be Muslim, but it is likely to become an issue of debate among aid professionals in the future.^37^ Perhaps the clearest guidance, however, is articulated by the National Zakat Foundation (NZF) in England where it is argued that, “The majority of scholars agree that Zakat is to be paid and received by Muslims only” (National Zakat Foundation 2021). Here, it is suggested that because *Zakat* is a religious obligation, this and the many other rules and regulations applied to *Zakat* are deemed to be directed to Muslims exclusively. On the other hand, other acts of charity by Muslims do not have the same rules and restrictions. Voluntary charity like *Sadaqah*, for example, can be donated to anyone and in any form, without prioritizing cash: “Hence, we encourage Muslims to also spend as much Sadaqah as possible to help both Muslims and non-Muslims (whether poor or not) in whatever form possible” (ibid).

## Conclusion

The challenge in turning an *ummatic* system into contemporary policy is whether the *umma* can be operationalized within the paradigm of the modern nation state. What is needed is not to destroy or abolish the model of a modern state, but to reform and develop it to be more humane in the light of the principles of the *umma*, especially with regard to policies of accepting refugees (whether they are Muslims or not), opening borders, providing support and assistance to them. The introducing of a modern application of the “*umma*” that can be implemented through the modern nation state has become pressing, not only to alleviate the suffering of refugees, but also to benefit the region and its development and to address its economic and political problems.

As demonstrated, there is a conceptual paradox between the *umma* and the modern nation-state, where the latter is by definition exclusionary while the *umma* is transnational and inclusionary. This phenomenon poses a problem for the sovereignty of nation-states. The *umma’*s supra-national claim over the people partially depends on its powers to decide whether or not the citizens of that nation-state may take in refugees. As Urquhart (2010, 7) recognised, “What is needed now is not to abolish national sovereignty but to reconcile it with the demands of human survival and decency in the astonishingly dangerous world we have absentmindedly created.” There are around a quarter of a billion migrants and 68 million refugees and displaced people around the world, and thus, a growing and urgent need to address the problematic of violations of the 1951 Refugee Convention that is increasing by parties to the convention. The ethical lessons of immigrants/refugees (muhājirūn) in Islamic history show how a state can be in harmony with *umma* without the state’s sovereignty being an obstacle from effectively responding to human needs. We can discover this meaning from how the early community of believers responded to the influx of refugees. The anṣār (literally, the supporters) gave complete refuge and integration to the exiled people who had been forced to leave their homes and much of their wealth in Makkah. This incident has been celebrated in the Qurʾan and held forth as a role model for future generations (Qurʾan, 59:9; Zaman, 2020, 8). As Derrida suggested, “the mastery of the house is ceded in order to transform both host and guest into something different—into neighbors or kin,” which means granting them complete protection and full integration (cited in Zaman, 2020, 8).

One of the major challenges of exploring and implementing the concept of a transnational *umma* in relation to migration and refuge (*hijra wa lujūʾ*) is the division of the Muslim World into multiple nation-states (Kamali 2011, 83). This paper suggests that there is a growing need to go beyond the contemporary state, which is characterized by strict border surveillance, military operations, and security wars. Various renowned scholars such as Richard Falk (2019) and Noam Chomsky (2015) have called for this. Here, the OIC is invited to play an effective role as a conduit to the *umma* functioning to prevent more lives being lost from false rationalisations, greed, and negligence. The collaboration between OIC and UNHCR can result in “enhancing refugee protection in OIC member states; multilateral cooperation, including burden and responsibility sharing to protect and assist refugees; and voluntary repatriation, as the most preferred durable solution for any refugee situation”^2^.

Many researchers have suggested that the OIC can play a pivotal role in producing a modern application of *umma*, ensuring that it is combatable with international refugee law and international humanitarian law, which has been amply demonstrated by Abou El-Wafa (2009). Wani (2019) connects with the idea that the OIC should play a role since there has been a profound hiatus in the Muslim world without the unifying ethic of *umma*.

The organization represents 57 countries of the Muslim world with the aim of establishing unity, solidarity, and brotherhood. OIC is the only international religio-political organization in the world and that too the largest intergovernmental organization after the United Nations Organization, representing every Muslim on the face of this earth. Because the political unity of Ummah was destroyed in 1923 with the fall of Ottoman Empire, Muslims around the world have been desperately searching for rebuilding the lost sense of unity and oneness so necessary for the fellow-feeling and faith of Muslims. (Wani, 2019, 1)

Until now, OIC is not operating as should to be, as noted by Thiam:

the relations between its members do not yet seem to have gone beyond a formal framework, although the Islamic Development Bank is developing interesting projects in a number of Muslim countries. A comprehensive policy, that is to say, a board and bold vision that can rise to the challenge of global geostrategic issues has still not emerged with sufficient clarity. (Thiam in Hareir & Mbaye 2011: 876)

The proposed propagation of *umma* as a role for the OIC is achievable, perhaps in concert with UNHCR, but not without the sincere will of the governments of the Islamic states and sincere international support of the international community to make real investments in the host countries of refugees. As pointed out by Jordan’s King Abdullah II.

Our holistic approach involves investment and growth, not just aid and relief. It prioritizes empowerment and self-sufficiency for refugees over a demeaning reliance on handouts. It brings us together as partners—countries, regions, the private sector, and international financial institutions—to build a new model for effective, sustainable action.

These investments under the auspice of OIC and UN are hoped to encourage more countries towards reforming their refugee polices and legislations and make it consistent with the *umma* principles and international law. This will offer a radical durable remedy in place of the current ad hoc and temporary responses in this troubled region of the world.

As discussed above, there is no explicit *umma* policy that operates in Muslim-Majority countries when it comes to migrants or refugees. Yet, it becomes apparent from the examples of Jordan, Turkey, and Bangladesh that there are implicit notions of *umma* that play some role in decision-making, especially in the context of refugee crises. This sense of moral commitment towards the *umma* is not an idea that can be relegated as anachronistic to the medieval period of history, assuming it has no contemporary relevance. Any Muslim today will not only understand what the *umma* is, but with a very real and perhaps emotional attachment to it. That is because it provides (even if idealized and unrealized) a sense of responsibility, belonging, and commitment to all Muslims.

The cases of Jordan, Turkey, and Bangladesh raise difficult questions, such as whether we can evaluate countries solely based on official policies, when it is apparent that a country may have no official refugee policy, yet still implement the idea of the *umma* or ad hoc notions of it. At the very least, there is a loose sense of moral commitment towards the *umma* that guides policy decision-making but perhaps only in crisis circumstances. Although Jordan made its own arrangements rather than sign the 1951 Refugee Convention, they have welcomed millions of refugees and granted citizenship to many. Thus, a nation may retain a moral commitment towards human rights from their understanding of *umma* (Abou Fadl 2005). And in this sense, the *umma* needs to be translated or operationalized into official or formal agreements under the auspices of OIC and UN.

This issue is raised within a context of questioning the effectiveness of international refugee law and conventions without a broader sense of moral and ethical duty. For example, while governments look for loopholes to circumvent the obligations of the refugee convention, the people in Syria have shown the power of moral obligation, prior to the violence that precipitated the crisis of 2015. In neighboring towns and villages of Syria, people opened their homes to fellow Syrians who were displaced by an on-going conflict. In 2013, out of 511,900 registered internally displaced people, and local families hosted 289,000 (Zaman 2016, 4). Individuals and families housed and fed refugees in an informal way because of their steadfast and morally binding commitment to inalienable human rights (ibid). This is a Kantian action of goodwill but also recognition that Islamic moral and religious values may be applied in refugee studies (Johnson 2018)*.*

Former colonial states also need to do more to mitigate refugee crises, with the understanding they have caused many of the conflicts that generate refugee flows today.

If you are worried about refugees coming to your shores—although I must point out that over 85% of the world’s refugees are hosted by developing countries—surely you should be in favour of international programmes that address the reasons why people flee their homes? (Espinosa, 2019).

Although the idea of a common humanity exists in the hearts and minds of many, it is very far from a political reality, despite the continuous attempts for the unity of nations by the United Nations. Richard Falk, the eminent emeritus professor of international law at Princeton suggests that “a sense of *global community*” which is offered by the Islamic idea of a non-territorial *umma* is the “keystone for a new world order that serves humanity by its dedication to the realization of human and global interests.” Hence, he argues, unless the UN overcomes the hegemony of national interest that is pursued by individual Member states, it will not be capable of “serving humanity in a historically resonant manner” (Falk 2019).

Finally, the revitalization of *umma* needs to be understood as a principle and consciousness beyond inter-, intra-, and supra-national structures. It is certainly an opportunity for the Muslim world especially in this strange historical moment where globalization is challenged and where there is a growing need to prepare for and protect asylum-seekers, refugees, and forced migrants generally. The idealization of the *umma* transcends the realpolitik of nation states and national interests. History tells us that “national interests” (which are not always well defined or justified) will hold sway when refugees are demonized as threatening the social and cultural fabric, depleting scarce resources, etc., even if such threats are untrue. Of course, some states are more powerful than others and can relegate concern and assistance to the poor and needy to other less powerful states, sometimes, paying them to do so. This is where human right organizations are invited to reveal violations, double standards, and exploitation by “naming and shaming” and offering remedies.

In the case of the UNHCR Refugee Zakat Fund, however, we are witnessing a very conscious, calculated *ummatic* aid, by means of religious imperatives, even though Muslims are the exclusive donors and recipients, largely because of their proportions in the populations of displaced. The access to such a boon in financial resources (even though still relatively small compared with overall Zakat funds) has its foundations, its motivations, in Islamic religiosity, law, and ethics. Of course, *Zakat* has always existed for Muslims, just as the *umma* has always existed for them, but that does not mean that non-Muslims cannot benefit from them—especially with Muslim scholars’ support for giving Zakat to non-Muslims in Muslim-majority countries (even if it is not yet being applied). Clearly, if Muslims want to donate charity voluntarily (*sadaqah*) to non-Muslims, they may do so and, as shown above, are encouraged to do so. Similarly, if the *umma* in principle can include non-Muslims, presumably they would request inclusion and/or be invited. How the *umma* could be replicated in a secular context may be a long discussion, debate, and negotiation. This will no doubt include the very notion that humanity and human rights already exists as an *ummatic* expression or understanding.

## Data Availability

Not applicable.

## Footnotes

1 While Lebanon cannot be classified as a Muslim state, it could certainly be considered, unofficially, as a Muslim-majority country.

2 Note that the refugee numbers quoted by UNHCR only relate to registered refugees. The actual numbers are significantly higher.

3 https://www.who.int/emergencies/rohingya-crisis-one-year-on (accessed June 04, 2019).

4 http://reporting.unhcr.org/node/2549?y=2019#year (accessed June 02, 2019).

5 https://www.asylumineurope.org/reports/country/turkey/access-labour-market-0 (accessed September 20, 2019).

6 http://www.arabnews.com/featured/news/805236

7 http://www.khaleejtimes.com/nation/government/uae-aid-to-syrian-refugees -is-the-highest.

8 The Qurʾan says: “When Moses arrived at the wells of Madyin, he found there a large group of men (*ummatan*) who were watering (their herds and flocks)” (Q 28: 23).

9 The Qurʾan states: “There is no creature that crawls upon the earth, nor bird that flies upon its wings, but that they are umam (communities) like yourselves. We have neglected nothing in the Book—and they shall be gathered unto their Lord in the end.” (Q 6: 39; Nasr 2017, 603).

10 For the different usages of term *umma* in the hadith see the references under “community” in Wensinck (1960, 47).

11 “No Jews live in the city, for they have been placed behind an inlet of the sea. [T]heir condition is very low... the Greeks hate the Jews, good and bad alike, and subject them to great oppression, and beat them in the streets...Yet the Jews are rich and good, kindly and charitable, and bear their lot with cheerfulness” (Benjamin in Haines n.d.).

12 In most periods of history, Sunna and Shia have coexisted in relative peace as members of one *umma* with no restrictions on their freedom of movement within *umma* lands. But in the late twentieth century, with the Iranian Shiite revolution and the frequent Shiite attempts to lead political and religious power in the Muslim world, violence erupted between Iran and Sunni states in many parts of the Middle East. This suggests that the reasons for their struggle whether contemporary or past (as between the Ottoman Empire and the Safavid) have less to do with religion than other factors such as disputes over power and influence (See Pruitt 2019). Noteworthy is that Iran, the largest Shiite political force, is a member state of the Organization of the Islamic Conference (OIC), whose members are mostly Sunni states. The OIC is perhaps the largest international organization through which the concept of an *umma* can be effectively achieved—if the OIC has been reformed and developed. Iran has contributed to this organization since 1969, participated in most of its conferences, and has had cultural activities with the Research Center for Islamic History and Arts in Istanbul (affiliated to OIC).

13 Capitulations, or *imtiyazat,* were commercial privileges given by the Ottoman courts to states such as Venice, Genoa, France, England, and Holland, from the fourteenth century and later included Poland, Naples, Florence, Ancona, Spain, Portugal, Catalan, Russia, Habsburg Austria, and Sicily. Capitulation privileges included the following provisions for non-Muslims:

- General security of person and property, including testamentary rights, freedom of worship, burial, and dress. repairs to ships, emergency rations, aid against attacks by corsairs, and abolition of the *lex naufragii* (Shipwreck and salvage law). Permission to address complaints to the head of the Muslim community.
- Extraterritoriality, including Consular jurisdiction. Consul’s salary and other exemptions.
- Abolition of collective responsibility. (Encyclopaedia of Islam, 1986, p. 1179).

14 It is instructive to cite here recent statements of the Italian Deputy Prime Minister, Luigi di Maio, in blaming France’s colonial behavior in Africa and how it results in forcing poor African migrants to flee their countries by running them as de facto colonies. He emphasized that “France has never stopped colonizing tens of African states … the EU should sanction France, and all countries like France, that impoverish Africa and make these people leave, because Africans should be in Africa, not at the bottom of the Mediterranean” (in Willsher, 2019).

15 While the first part (nation) of the modern nation-state implies a connotation to a people who maintain common roots in terms of language, culture, religion, etc., the second part (state) refers to the fact that each nation has to be represented in a single state that reflects an identity and harmony among its members or citizens. This is consistent with “the classic European assumption that nations must have states and that, if possible, each state must have one homogenous nation” (Evans and Newnham 1998, 28). However, the existing reality of nation-states is not always consistent with this definition. Some nation-states have two nations within their territories, such as Bosnia, Herzegovina and Myanmar (Rosenberg 2019). Many nations are stateless, such as the Kurds in Iraq, Iran, Syria, and Turkey (Tallini 2007, 1). They do not belong to any state that is recognized by the international community (Payind and McClimans 2016, 91). This reality gives rise to violent conflicts in an attempt to gain recognition under the principle of “self-determination.” On the other hand, a single nation may be divided into many states, such as the Arabs who are “living in more than twenty sovereign states. They all belong to one Arab nation that is politically organized in a number of sovereign entities” (Köchler 1993, 1).

16 Nativism is a policy that privileges the interests of dominant citizens over newly arrived immigrant citizens (see Collin 2004, 170).

17 It is useful to distinguish between these three key terminologies: nation, nationalism, and patriotism. According to the Oxford English Dictionary (1989, vol.10), the nation is “an extensive aggregate of persons so closely associated with each other by common descent, language, or history, as to form a distinct race of people, usually organized as a separate political state and occupying a definite territory”. This is a standard definition but of course nation states were sometimes formed quite arbitrarily or for colonial purposes, such as the Sykes-Pico agreement dividing the Middle East. By contrast, nationalism refers to an attitude or policy that identifies one’s own country as of primary interest compared with the interests of other countries. Attitudinally, nationalism it may imply a sense of superiority as well as independence from other states. Thus, the nation is more relevant to the demography of the people, while nationalism is more relevant for forming a political as well as cultural or ethnic identity. Patriotism means a deep commitment and loyalty to one’s country of citizenship. Patriotism can become altruistic to the extreme extent of fighting and dying for it, in terms of military service and defense. While patriotism has a positive connotation in the passionate commitment of individuals, nationalism is often viewed as negative because of extremist chauvinistic political movements against “foreigners” who are denied their sense of belonging that is often based on ethnic and religious differences (see: https://www.dictionary.com/e/patriotism-vs-nationalism).

18 As the matter of fact, before the existence of nation-states “there were hardly any traces of nationalism, either in the Islamic world or in Europe” (Al-Ahsan 1992, 30).

19 In his talk at a ceremony marking the 100th anniversary of the end of World War I, French President Macron took the opportunity to slam President Trump’s “America First” nationalism. Macron stated, “Patriotism is the exact opposite of nationalism: Nationalism is a betrayal of patriotism. By putting our interests first, with no regard for others, we erase the very thing that a nation holds dearest and the thing that keeps it alive: its moral values” see; https://thepeninsulaqatar.com/article/11/11/2018/Nationalism-is-a-betrayal-of-patriotism-Macron.

20 Despite the continuous endeavors of the UN in promoting a sense of global community and disseminating the universalizing values, nationalism still threatens both domestic and international communities. In her speech H.E. Mrs. María Fernanda Espinosa, President of the 73rd Session of the UN General Assembly in April 2019 warned, “We see it—nationalism—in the challenges to regional cooperation—in Europe, for instance, but also in Latin America … it is not an easy time for the region” (Espinosa 2019).

21 Securocrats refers to “military or police officers who have the power to influence government policy” (Collins English Dictionary, 2014). Thus, it denotes powerful and influential security services of governments. In the same vein “securocratic” borders, as delineated by Allen Feldman means the kind of policing when national and other forms of physical borders disappear or are no longer enforceable. New boundary systems emerge beginning with poverty stricken urban districts and replicated throughout cities and country areas. These securocratic “borders” “are virtual, mediatized, such as electronic, biometric, and digital surveillance nets” and the policies “are focused on countering imputed territorial contamination and transgression/ “terrorist”, demographic, and biological infiltration” (Feldman, 2006, 331–335). In sum, as Halper (in Deutsch, 2019) notes, it is a process by security forces to “securitize everything” and that is increasingly made possible by contemporary. surveillance technology. The gravity of these policies increases when they are “left to securocrats and then there is a danger of a drift to a police state,” (Sir Peter Fahy, chief constable of Greater Manchester Police, in *The Independent*, Saturday 06 December 2014. https://www.independent.co.uk/news/uk/politics/britain-could-drift-towards-a-police-state-says-one-of-britain-s-top-police-officers-9908113.html).

22 At the same time, of course, as Weber identified, the state authority, party, or government is only recognized when it successfully demonstrates that it has a monopoly over the legitimate use of violence within its territory (Weber, 1946/1919).

23 The effect upon the solidarity of the European Union from the impending Brexit is too early to tell.

24 It is interesting to apply this to the current criticism and blockade against Qatar by the Saudi-led coalition with UAE, Bahrain, and Egypt. They demand that Qatar sever ties with the Muslim Brotherhood and stop protecting them in Qatar. Like most countries of the world, including the UK, the Muslim Brotherhood are not considered a terrorist group, but individual members can be given refugee status and protection under international refugee law.

25 Mashrek (or Mashreq) is comprised of the Arab states: Bahrain, Egypt, Iraq, Jordan, Kuwait, Lebanon, Oman, Palestine, Qatar, Saudi Arabia, Sudan, Syria, the United Arab Emirates, and Yemen.

26 The *anṣār*, or “The Helpers”, were the locals in Medina who assisted the Prophet Mohammed and his followers, who were known as the *muhājirūn* when they fled from Mecca.

27 See*: “Helping Rohingya is Turkey’s one of main foreign policy objectives” *https://www.aa.com.tr/en/asia-pacific/-helping-rohingya-is-turkeys-one-of-main-foreign-policy-objectives/1952518

28 *Arab News*, 8 December 2020: https://www.arabnews.com/node/1774306/world

29 Memorandum (Jordan), 107, article 5.

30 https://kingabdullah.jo/en/speeches/during-%E2%80%9Csupporting-syria-and-region-conference%E2%80%9D

31 See*: In Jordan, supporting one of the biggest refugee-focused operations ever:*
https://www.unv.org/Success-stories/Jordan-supporting-one-biggest-refugee-focused-operations-ever

32 Interview with the Bangladesh Consul in Qatar.

33 https://reliefweb.int/sites/reliefweb.int/files/resources/GULFreportdesign14.pdf (accessed November 09, 2019).

34 https://reliefweb.int/sites/reliefweb.int/files/resources/GULFreportdesign14.pdf (accessed November 09, 2019).

35 *UN News*, “Islamic law major influence on modern global refugee law,” 22 June 2009. https://news.un.org/en/story/2009/06/304232-islamic-law-major-influence-modern-global-refugee-law-un-study

36 Kidwai and Zidani (2020: 51), suggest it is 6.5%.

37 We are grateful to anonymous reviewer #1 for the insightful comments on this issue and for suggesting an elaboration of *Zakat* for this paper.
